# Effects of *Escherichia coli* Nissle 1917 and Ciprofloxacin on small intestinal epithelial cell mRNA expression in the neonatal piglet model of human rotavirus infection

**DOI:** 10.1186/s13099-016-0148-7

**Published:** 2016-12-13

**Authors:** Francine C. Paim, Stephanie N. Langel, David D. Fischer, Sukumar Kandasamy, Lulu Shao, Moyasar A. Alhamo, Huang-Chi Huang, Anand Kumar, Gireesh Rajashekara, Linda J. Saif, Anastasia N. Vlasova

**Affiliations:** 1 Food Animal Health Research Program (FAHRP), The Ohio Agricultural Research and Development Center, Veterinary Preventive Medicine Department, The Ohio State University, 1680 Madison Avenue, Wooster, OH 44691 USA; 2 Hillman Cancer Center, University of Pittsburgh, 4200 Fifth Ave, Pittsburgh, PA 15260 USA; 3 Genomics and Systems Biology, Bioscience Division, Los Alamos National Laboratory, Los Alamos, NM 87545 USA

**Keywords:** Probiotic, Antibiotic, Commensal microflora, Gnotobiotic piglets, Human rotavirus, Intestinal epithelial cell-specific genes

## Abstract

We evaluated the effects of the probiotic *Escherichia coli* Nissle 1917 (EcN) and the antibiotic Ciprofloxacin (Cipro) on mRNA expression of intestinal epithelial cells (IEC) in gnotobiotic (Gn) piglets colonized with a defined commensal microflora (DMF) and inoculated with human rotavirus (HRV) that infects IECs. We analyzed mRNA levels of IEC genes for enteroendocrine cells [chromogranin A (CgA)], goblet cells [mucin 2 (MUC2)], transient amplifying progenitor cell [proliferating cell nuclear antigen (PCNA)], intestinal epithelial stem cell (SOX9) and enterocytes (villin). Cipro treatment enhanced HRV diarrhea and decreased the mRNA levels of MUC2 and villin but increased PCNA. These results suggest that Cipro alters the epithelial barrier, potentially decreasing the numbers of mature enterocytes (villin) and goblet cells secreting protective mucin (MUC2). These alterations may induce increased IEC proliferation (PCNA expression) to restore the integrity of the epithelial layer. Coincidental with decreased diarrhea severity in EcN treated groups, the expression of CgA and villin was increased, while SOX9 expression was decreased representing higher epithelial integrity indicative of inhibition of cellular proliferation. Thus, EcN protects the intestinal epithelium from damage by increasing the gene expression of enterocytes and enteroendocrine cells, maintaining the absorptive function and, consequently, decreasing the severity of diarrhea in HRV infection.

## Background

Rotavirus infects intestinal epithelial cells (IECs) causing villous atrophy and malabsorptive diarrhea [1] and is a major cause of gastroenteritis in children worldwide, leading to more than 500,000 deaths annually [2]. Gnotobiotic (Gn) piglets are used as a model to study virulent human rotavirus (VirHRV) infection due to their susceptibility to diarrhea and similarities in gastrointestinal physiology, micro- and macronutrient metabolism, and immunity when compared to human infants [3]. In developing countries, the two currently licensed live attenuated HRV vaccines show reduced efficacy due to multiple factors, including micronutrient deficiencies, intestinal dysbiosis, and genetic variability among dominant RV strains [4, 5]. Indiscriminate use of antibiotics aggravates intestinal dysbiosis and is frequently associated with persistence of HRV-induced diarrhea [6]. Therefore, alternative strategies are important to alleviate infectious diarrhea and/or enhance oral vaccine efficacy.

Commensal microbiota and probiotics interact with host metabolic activities and immune responses to protect the intestinal epithelium against enteric pathogens [7, 8]. *Escherichia coli* strain Nissle 1917 (EcN) is a Gram-negative probiotic that acts as a potent immunostimulant of the host immune system [9], and its immunomodulatory and anti-inflammatory effects alleviate HRV-induced diarrhea in Gn piglets [10, 11]. However, the effects of EcN on IEC dynamics during HRV infection have not been studied. It is well established that intestinal epithelial stem cells (IESCs) respond to intestinal damage, maintaining a dynamic balance between the stem cell progenitors, secretory (goblet, enteroendocrine) cells and enterocytes [12]. Here, we evaluated the effects of EcN and/or Ciprofloxacin (Cipro), a fluoroquinolone antibiotic commonly used in infants and children to treat infectious diarrhea, on mRNA expression profiles of IEC specific genes for enteroendocrine cells [chromogranin A (CgA)], goblet cells [mucin 2 (MUC2)], transient amplifying progenitor cell [proliferating cell nuclear antigen (PCNA)], intestinal epithelial stem cell (SOX9) and enterocytes (villin) during HRV infection of Gn piglets colonized with a defined commensal microflora (DMF). The DMF, similar in composition to modified Schaedler’s flora used in mice, consists of seven bacterial species of swine origin (*Bifidobacterium adolescentis*, *Bifidobacterium longum*, *Bacteroides thetaiotaomicron*, *Enterococcus faecalis*, *Lactobacillus brevis*, *Streptococcus bovis* and *Clostridium clostridioforme*) [13].

Comparative assessment of the expression levels of IEC genes should reflect intestinal crypt dynamics in response to Cipro and/or EcN treatments in the context of HRV infection of IECs. The complexity of the intestinal microenvironment of conventional neonatal piglets complicates delineation of the specific effects of probiotics and antibiotics. To establish a simplified model of commensal microbiota, we used the DMF bacterial cocktail derived from the gut of healthy pigs. Like individual probiotic strains, each DMF bacterial strain colonizes Gn pigs after a single dose and influences early maturation of neonatal immune responses [14–17] which otherwise are naïve and functionally immature compared to adults [18, 19].

## Methods

### Experimental design

This study was approved by the Institutional Animal Care and Use Committee at Ohio State University (protocol #2010A00000088). Cesarean-derived Gn piglets from sows (Landrace × Yorkshire × Duroc cross-bred) were maintained in sterile isolators as described previously [20]. All piglets were colonized orally at 7 days of age with DMF with 10^5^ colony-forming units (CFU) of each bacteria/piglet. DMF were kindly provided by Dr. David Francis from South Dakota State University, USA.

Piglets were randomly assigned to 4 groups: DMF + VirHRV (n = 6), DMF + Cipro + VirHRV (n = 6), DMF + EcN + VirHRV (n = 3) and DMF + Cipro + EcN + VirHRV (n = 4). The piglets were orally treated or untreated with Cipro (60 mg/day) and/or EcN (10^5^ CFU/piglet daily) at post bacterial colonization days (PBCD) 8–13. EcN inoculum was prepared as described previously [10]. All piglets were challenged with VirHRV at a dose of 2 × 10^6^ fluorescent-forming units (FFU) per piglet at PBCD 14. Post-VirHRV challenge, rectal swabs were collected to assess HRV shedding by cell culture immunofluorescence infectivity (CCIF) assay and record fecal scores to assess the severity of diarrhea as described previously [21]. All piglets were euthanized by electrocution following anesthesia at PBCD 35/post-VirHRV challenge day (PCD) 21 and mid-jejunum (10 cm) was collected to isolate IECs.

### Isolation of IECs

The IECs were isolated from jejune (middle gut) using a modified protocol adapted from Pan et al. [22]. Briefly, jejunum was cut into small pieces (1 cm) and placed in a 50 ml tube with 20 ml of Hanks balanced salt solution (Gibco BRL, Gaithersburg, MD, USA) containing 5% fecal bovine serum (FBS) (Sigma-Aldrich, St. Louis, MO, USA) and 2.5 mM EDTA (Sigma-Aldrich, St. Louis, MO, USA). The tissue was processed twice in an orbital shaker at 300 RPM for 15 min and the resulting cell suspension was filtered through a metal cell strainer. The IECs were spun down at 500 × g for 10 min at 4 ^°^C and the pellet was resuspended in RPMI1640 (Gibco BRL, Gaithersburg, MD, USA) enriched with 8% FBS (Sigma-Aldrich, St. Louis, MO, USA), 2 mM l-glutamine (Gibco BRL, Gaithersburg, MD, USA), 0.1 mM nonessential amino acids (Gibco BRL, Gaithersburg, MD, USA), 1 mM sodium pyruvate (Gibco BRL, Gaithersburg, MD, USA), 20 mM HEPES (Gibco BRL, Gaithersburg, MD, USA), 100 g of gentamicin (VetOne, Boise, ID, USA) per ml, and 10 g of ampicillin (Gibco BRL, Gaithersburg, MD, USA) per ml (E-RPMI). The viability and numbers of IECs were determined by the Trypan Blue exclusion method. IECs were stored at −80 °C in 500 ul of RNA later tissue collection buffer (Life technologies, Carlsbad, CA, USA) until further analysis.

### Extraction of RNA

Total RNA from IECs was extracted using Direct-Zol RNA Miniprep (Zymo Research, Irvine, CA, USA) according to the manufacturer’s instructions. The RNA concentrations and purity were measured using NanoDrop 2000c spectrophotometer (Thermo Scientific, Wilmington, DE, USA).

### Real-time quantitative RT-PCR (qRT-PCR)

qRT-PCR was performed using equal amounts of total RNA (75 ng) with Power SYBR Green RNA-to-CT 1 step RT-PCR kit (Applied Biosystems, Foster, CA, USA). The primers for CgA, MUC2, PCNA, SOX9, villin and β-actin were based on previously published data [23–25]. Relative gene expression of CgA, MUC2, PCNA, SOX9 and villin were normalized to β-actin and expressed as fold change using the 2ΔΔCt method [26].

### Statistical analyses

Mean days to onset of virus shedding, mean duration of virus shedding, average peak virus shedding titer and mean duration of diarrhea were analyzed by the Kruskal–Wallis rank-sum test. Mean cumulative fecal scores were analyzed by the area under the curve method as described previously [10]. A nonparametric *t* test was used to detect significant differences (P < 0.05) in the relative mRNA levels between the treatments and control group. Statistical analyses were performed by using GraphPad Prism 5 software (Graph Pad Software) for relative mRNA levels and SAS 9.4 (SAS Institute Inc. Cary, NC) for shedding titers and diarrhea scores.

## Results

### Cipro increases diarrhea severity, while EcN induces protection against HRV-induced diarrhea in Cipro treated piglets

**Fig. 1:**
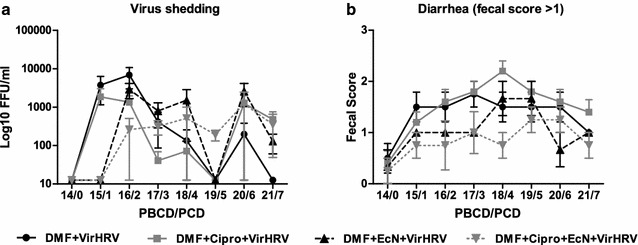
Virus shedding (**a**) determined by CCIF and expressed as FFU/ml and duration of diarrhea (**b**) determined by number of days with fecal score >1 (fecal consistency was scored as follows: 0 = normal, on diarrhea: 1 = pasty/semiliquid, 2 = liquid) in DMF + VirHRV (n = 6), DMF + Cipro + VirHRV (n = 6), DMF + EcN + VirHRV (n = 3) and DMF + Cipro + EcN + VirHRV (n = 4) groups

Fecal virus shedding was confirmed for all DMF-colonized, VirHRV-challenged piglets (Fig. 1a). Mean days to onset of shedding were significantly longer in DMF + Cipro + EcN + VirHRV compared to DMF + Cipro + VirHRV piglets (Fig. 1A). Mean duration of diarrhea was longer in DMF + Cipro + VirHRV compared with DMF + VirHV piglets (Fig. 1b).

**Fig. 2:**
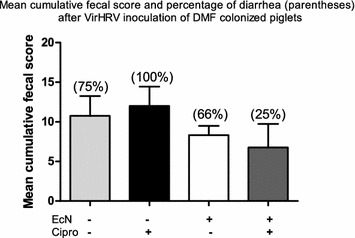
Mean cumulative fecal score (daily fecal scores from PCD 1–7/n) to assess the severity of diarrhea and percentage of diarrhea in DMF + VirHRV (n = 6), DMF + Cipro + VirHRV (n = 6), DMF + EcN + VirHRV (n = 3) and DMF + Cipro + EcN + VirHRV (n = 4) groups. Means with different letters (**a**, **b**) in the same column differ significantly (determined by the Kruskal–Wallis rank sum test, P ≤ 0.05)

Cipro treatment resulted in a 25% increase in the proportion of diarrheic piglets post-VirHRV challenge when compared to DMF-colonized, non-Cipro treated animals. Notably, EcN-treatment greatly reduced the percentage of piglets with diarrhea in both Cipro and non-Cipro-treated, DMF-colonized groups. Furthermore, DMF + EcN + VirHRV and DMF + Cipro + EcN + VirHRV groups had significantly lower mean cumulative fecal diarrhea scores compared to groups not treated with EcN (Fig. 2).

### Contrasting effects of Cipro and EcN treatments on the gene expression by goblet cells, enterocytes, enteroendocrine, transient amplifying progenitor and IESCs of HRV infected piglets

**Fig. 3:**
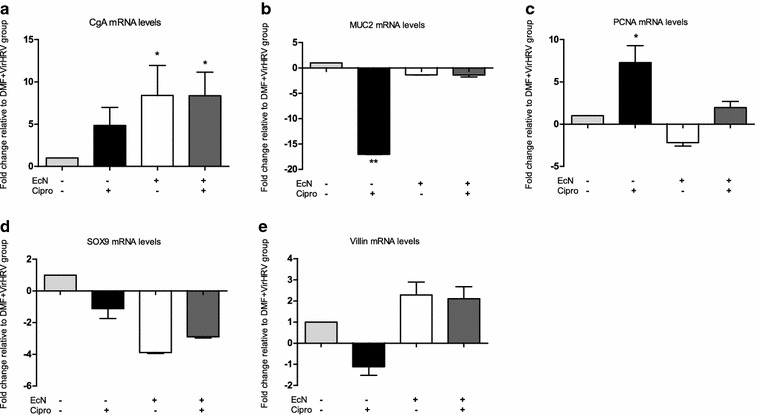
Relative mRNA levels of CgA (**a**), MUC2 (**b**), PCNA (**c**), SOX9 (**d**) and villin (**e**) in DMF + Cipro + VirHRV (n = 6), DMF + EcN + VirHRV (n = 3), DMF + Cipro + EcN + VirHRV (n = 4) groups measured by RT-PCR, normalized to β-actin gene and expressed as fold change relative to the DMF + VirHRV group (n = 6), which was normalized as 1. Graphs represent means ± SEM. (^*^P < 0.05, **P < 0.01, relative to DMF + VirHRV group)

Gene expression levels of CgA, MUC2, PCNA, SOX9 and villin were assessed. The relative mRNA levels of CgA were significantly increased in EcN groups with or without Cipro (DMF + EcN + VirHRV, DMF + Cipro + EcN + VirHRV) when compared with DMF + VirHRV piglets (Fig. 3a). Cipro treatment (DMF + Cipro + VirHRV) significantly downregulated MUC2 while upregulating PCNA mRNA levels when compared with the DMF + VirHRV group (Fig. 3b and c). Gene expression of SOX9 was downregulated in all groups in comparison with DMF + VirHRV, but more so in the EcN-treated groups (DMF + EcN + VirHRV, DMF + Cipro + EcN + VirHRV, Fig. 3d). Cipro treatment decreased the relative mRNA levels of villin in DMF + Cipro + VirHRV animals; however, EcN-treatment (DMF + EcN + VirHRV, DMF + Cipro + EcN + VirHR) upregulated villin when compared with the DMF + VirHRV piglets (Fig. 3e).

**Fig. 4:**
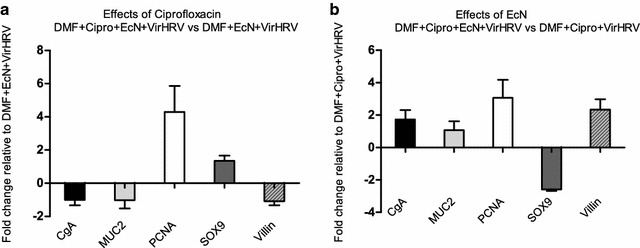
Effects of Cipro (**a**) on mRNA levels of CgA, MUC2, PCNA, SOX9 and villin in DMF + Cipro + EcN + VirHRV (n = 4) expressed as fold change relative to DMF + EcN + ViHRV (n = 3) piglets. Effects of EcN (**b**) on mRNA levels of CgA, MUC2, PCNA, SOX9 and villin in DMF + Cipro + EcN + VirHRV (n = 4) piglets expressed as fold change relative to DMF + Cipro + ViHRV (n = 6) group. Graphs represent means ± SEM

Additional effects of Cipro on IECs were observed. Relative mRNA levels of CgA, MUC2 and villin decreased, whereas gene expression of PCNA and SOX9 increased in DMF + Cipro + EcN + VirHRV in comparison with DMF + EcN + VirHRV piglets (Fig. 4a).

The modulatory effects of EcN on IEC gene expression levels were further demonstrated. Increased relative expression of all genes (with the exception of SOX9) was observed in DMF + Cipro + EcN + VirHRV compared with DMF + Cipro + VirHRV piglets (Fig. 4b).

## Discussion

In this study, we investigated the effects of the probiotic EcN, the antibiotic Cipro and their combined effects on VirHRV infection and IEC dynamics in DMF-colonized, VirHRV-challenged piglets. Cipro treatment increased the severity of VirHRV diarrhea as demonstrated by an increased incidence of diarrhea and mean fecal cumulative diarrhea score. Cipro treatment in healthy humans decreased the taxonomic richness, diversity, and evenness of gut microbiota [27] as well as decreasing these parameters in Gn pigs in our study (Huang et al., unpublished).

We observed a negative effect of Cipro on MUC2 mRNA levels suggesting decreased mucin secretion from goblet cells. In agreement with our results, antibiotic metronidazole decreased MUC2 production from goblet cells suggesting a potential depletion of the mucus layer, predisposing the host to enteric infection [28]. In addition, our results are supported by other studies that noted changes in the gut microbiota composition and intestinal homeostasis leading to decreased MUC2 secretion [29].

We found that Cipro treatment during HRV infection significantly upregulated mRNA levels of PCNA. This is likely due to increased intestinal damage that stimulates increased epithelial proliferation. Furthermore, Cipro treatment decreased the gene expression of villin during HRV infection. Similarly, other researchers observed downregulation of other enterocyte genes including lactase, liver fatty acid-binding (L-FABP) and sodium-dependent glucose transporter 1 (SGLT1) in the small intestine of mice during murine RV infection [30]. Our results suggest a negative feedback mechanism between numbers of mature enterocytes and the levels of IEC proliferation [31]. We conclude that HRV infection might induce apoptosis of mature enterocytes as observed by others for murine RV [30] and, consequently, stimulate IEC proliferation. However, these cells are replaced by less differentiated enterocytes, leading to defective absorptive function and increased secretory HRV diarrhea [30, 32].

Probiotics provide a physical barrier to block pathogen entry into IECs [8], thereby reducing diarrhea duration and enhancing enterocyte proliferation and villus repopulation [33]. In this study, the DMF + Cipro + EcN + VirHRV piglets had decreased mean cumulative fecal diarrhea scores and diarrhea prevalence compared with DMF + Cipro + VirHRV piglets. Our results suggest that EcN protects the intestinal epithelium from damage as confirmed by an increase in mRNA levels of CgA, and villin in addition to decreased diarrhea severity. Similarly, in a double-blind trial, EcN conferred beneficial effects by reducing diarrhea in young children [34]. EcN has also been used to alleviate the severity of diarrhea in gastrointestinal diseases such as ulcerative colitis [35], inflammatory bowel disease [36] and Crohn’s disease [37]. Our study corroborated our previous findings that demonstrated EcN protective effects against HRV [10, 11]. However, from our results we can only suggest a beneficial effect of EcN on IEC gene profile expression during HRV infection. Further evaluation of the intestinal epithelial layer and total cell numbers need to be confirmed by histopathological examination and/or immunohistochemistry.

The upregulation of CgA in EcN-treated piglets could be reflective of enhanced protection of the intestinal barrier. Other studies have shown that enteroendocrine cells are activated after treatment with probiotics [38]. Enteroendocrine cells are regulated by Notch signaling and produce hormones that control various functions such as glucose metabolism, exocrine pancreatic secretion and repair of intestinal epithelium [39]. In addition, we observed a decrease in expression of the stem-cell specific-gene SOX9 in the EcN-treated groups. SOX9 plays an important role in control of the proliferative capacity of stem cells to replenish different lineages of IECs [40]. A decrease in SOX9 gene expression could be explained by the ability of probiotics to modulate cellular proliferation by Wnt signaling inhibition [41]. However, further studies are needed to elucidate the numbers of intestinal stem cells before and after treatment to clarify this question.

Our study demonstrated that EcN increases the mRNA levels of the enterocyte-specific gene villin. These results suggest that EcN modulates the effects of HRV and Cipro by increasing the villin gene expression of enterocytes and repairing/restoring functional enterocytes, resulting in increased barrier and absorptive functions during HRV-induced diarrhea. Additionally, treatment with the probiotic LGG increased the number of villus cells in the jejunum of gnotobiotic rats [42].

In conclusion, our findings suggest that Cipro can enhance RV pathogenesis by disrupting intestinal homeostasis, affecting the IEC dynamics and potentiating the severity of diarrhea. To our knowledge, our current study is the first to demonstrate the beneficial effect of EcN in increasing the expression of the villin gene during HRV infection. Thus administration of the probiotic EcN protects the intestinal epithelium and alleviates diarrhea during VirHRV infection of DMF-colonized Gn piglets. Further studies are necessary to investigate the role of EcN in enhancing rotavirus vaccines efficacy in conditions where children are exposed to antibiotics and malnutrition.
